# Editorial: Advances in Sport Science: Latest Findings and New Scientific Proposals

**DOI:** 10.3389/fpsyg.2022.891906

**Published:** 2022-04-28

**Authors:** Rubén Maneiro, José Luís Losada, Claudio A. Casal, Sophia Papadopoulou, Hugo Sarmento, Antonio Ardá, Xavier Iglesias, Mario Amatria

**Affiliations:** ^1^Department of Science of Physical Activity and Sport, Pontifical University of Salamanca, Salamanca, Spain; ^2^Department of Social Psychology and Quantitative Psychology, University of Barcelona, Barcelona, Spain; ^3^Department of Science of Physical Activity and Sport, Catholic University of Valencia “San Vicente Mártir”, Valencia, Spain; ^4^Faculty of Physical Education and Sport Science, Aristotle University of Thessaloniki, Thessaloniki, Greece; ^5^Faculdade de Ciências do Desporto e Educação Física, Universidade de Coimbra, Coimbra, Portugal; ^6^Department of Physical and Sports Education, University of A Coruña, A Coruña, Spain; ^7^Institut Nacional d'Educació Física de Catalunya (INEFC). University of Barcelona, Barcelona, Spain

**Keywords:** editorial, sport science, methodology, performance analysis, high performance

Although sport as an activity has been practiced for much of modern history, sports sciences were not considered a discipline of academic tradition until the 20th century (Fernández and García, 2018). The purpose and function of sport sciences are to investigate questions about motor behavior and performance, which must be solved on a scientific basis.

According to data from PubMed, scientific research on sport sciences has increased in the last 10 years. Specifically, it is possible to affirm that more scientific studies were published in the 2010–2020 decade than in the entire previous period (1945–2009) (Maneiro, 2021). This brings us closer to the idea that this area of knowledge is in full expansion and apogee, in which sports scientists have a fundamental role.

Analyzing more specifically the different fields of study, it is possible to affirm that some fields have more robust growth, while in others their growth is more moderate. Specifically, areas such as rehabilitation, exercise, or biomechanics show very notable growth, while others such as sports injuries, motor behavior analysis, performance analysis, or strength training show less notable growth (González et al., 2018).

This special Research Topic entitled “*Advances in Sport Science: Latest Findings and New Scientific Proposals*” began with a double objective: on the one hand, to offer a space where scientists can continue to delve into the most consolidated scientific disciplines; and on the other hand, to open a path where those areas that still need more research could have a place. As a result, the great impact it has had on the community is noteworthy, to the extent that 27 articles have been published by 130 authors, and with a total global impact of almost 61,000 visits from multiple different countries, which has increased and improved knowledge on the following topics: performance analysis in individual and team sports (15 articles), the impact of COVID-19 on performance (3 articles), executive functions and physical fitness at an early age (3 articles), physical activity in older people (1 article), and psychological profiles in performance athletes (6 articles).

## Overview of Contributions

The analysis of sports performance has been the subject that research has most concentrated on. This is no coincidence, since the abundance of currently existing sports disciplines demands research that results in potential recommendations with empirical support. More specifically, football is the sport that has been the subject of most research, specifically with five studies, and they have focused on the comparison of goal scoring patterns in the main European leagues (Li and Zhao), the peak performance age of top-level soccer players (Oterhals et al.), differences in technical and space management actions in three UEFA Champions League finals (Amatria et al.), the analysis of one of the most important rules of the game in soccer, such as offside (Zhao), and the opinion of game analysts on different aspects of the game (Aguado-Méndez et al.). All these studies have allowed us to increase our knowledge about different variables that may be modulating success in performance football. The analysis of other team sports, such as volleyball, assessed the efficiency of the training process with different methodologies (Fernández-Echevarría et al.), the influence of the yips (psycho-neuromuscular disorder characterized by involuntary movements that disrupt the execution of automatic fine motor behavior) in college baseball players (Aoyama et al.), the analysis of ultimate frisbee from different criteria such as where more passes are made and what behaviors differentiate the winning teams from the losers (Lam et al.) and, finally, it has been shown that in sports such as rowing (Gavala-González et al.) there is a relationship between the academic record of athletes and sports performance. Specifically, rowers who obtain better academic grades have higher levels of involvement in the tests, and therefore better sports results.

On the other hand, individual sports have also had a special space within the topic. An example is the study by León-Guereño et al., where they analyze the influence of different variables such as age, sex, or marital status on the motivation toward endurance sports such as athletics; on the other hand, badminton (an individual or dual sport) has also had a special mention with two articles: the study by Torres-Luque et al. has proposed the design and validation of an observation instrument for the analysis of technical and tactical behaviors in badminton; and the study by Guo et al., where they analyzed the effect of combined balance and plyometric training on change of direction performance in a 6-week program. Finally, the work of Núñez-Barriopedro et al., with a sample of 682 karate fighters, analyzed the strategies of karate federations to attract and retain competitors through variables such as happiness and how it modulates performance.

Of course, this special Research Topic could not ignore the influence of the COVID-19 pandemic on both the training and sports performance of athletes. In this sense, three studies have focused their efforts on the impact not only of COVID, but also the performance within the bubble in world championships. More specifically, the work of Gentile et al. analyzes how the pandemic influenced different psychological variables such as stress and the ability to fall asleep in the “bubble” at the 2020 World Samboo Championships; on the other hand, the work of McLean et al., using the STS (sociotechnical systems) theory shows that the sports teams of the Australian Football League can take advantage of the circumstances of the pandemic to improve the efficiency of their departments; and finally, the study by Bobo-Arce et al. collects 302 interviews with rhythmic gymnastics coaches from 26 different countries, where they conclude that although gymnasts continued training during confinement, almost 3 out of 4 reported some abandonment of sports practice, also proposing training advice for future lockdowns.

A novel feature of this Research Topic has been the attention paid to early ages from two perspectives: the assessment of physical fitness in boys and girls, as well as their related executive functions. In this sense, the work of Escolano-Pérez et al. examined the influence of early environmental variables such as the type of feeding or the mode of delivery and some biological variables (sex and age) on pre-school motor skills, finding notable differences between them based on sex and type of delivery, among others. The study by Veraksa et al., with a sample of 261 boys and girls from 5 to 6 years of age, tried to identify the possible different levels of physical fitness among them, in addition to an analysis of their executive functions. Lastly, the ambitious study by Iglesias-Soler et al., entitled “Percentiles and principal component analysis of physical fitness from a big sample of children and adolescents aged 6–18 years: the DAFIS Project” with more than 15,000 young people from Galicia, concluded that the physical condition was better in boys than in girls, and that the distribution of fat mass and muscle performance had a high proportion of variation in physical fitness. On the other hand, physical activity in older people has also had a special place in this Research Topic with the article “Physical activity and life satisfactions: an empirical study in a population of senior citizens” by Wöbbeking et al., where, with a sample of 300 older subjects, the influence of various sociodemographic variables (age, sex, institutionalization, and level of education) on the performance of physical activity is analyzed, demonstrating that people with a higher level of education present differences in physical and motivational reserves, and that the latter affect healthy cognitive aging.

Another novel research focus in the current Research Topic has been the inclusion of studies on eye fixation and the “quiet eye” effect. The study of Dahl et al. monitored eye behavior and vision in a similar context to e-sports in more than 2,600 trials, stating that increased cognitive load delayed the onset of gaze fixation, and that the duration of the last fixation before a motor action predicted performance outcome.

Finally, the psychological approach in performance analysis was addressed in five different studies, and four different sports. In the first place, the study by Uriarte et al. focused on judo, and found that the performance indicators that made the greatest difference were psychological aspects such as motivation, stress, and team cohesion, with the motivation variable being the most important for success. On the other hand, there have been two studies that have focused on psychological aspects in archers. The study by Wu et al. studied the effects of a mindfulness intervention program (mindfulness-based peak performance, MBPP) on sports performance in 23 archers, showing that the MBPP program significantly improved shooting performance, multiple cognitive functions, and the level of negative ruminations decreased significantly. On the other hand, the investigation of Li et al. (43 archers and shooting specialists were analyzed) intended to investigate the relationship between the level of experience and interoceptive attention capacity in shooting and archery, concluding that elite athletes outperformed amateurs in different aspects and variables considered. Likewise, Peng et al. studied the relationship between competitive cognitive anxiety and motor performance in Chinese college basketball players, concluding that ego and task orientations and the “goal profile” moderate the relationship between competitive anxiety and motor performance. Finally, in the work of Mitic et al., the differences in the psychological profiles of elite and non-elite athletes are analyzed, stating that the former are characterized by a positive score toward self-efficacy, emotionality, low negative past time perspective, emotional competence, and future time perspective, while the latter have completely opposite features. Finally, in the study entitled “Use of Stroop Test for sports psychology study: cross-over design research” by Takahashi and Grove, they used the Stroop psychological test to investigate the benefits of exercise on cognitive function, finding that there was no significant effect on the exercise mode for both Stroop and reverse Stroop interference.

## Future Lines of Research

In view of the large number of published articles, all of them of high methodological and substantive quality, the present Research Topic has responded to those objectives that the editors have aspired to: to help increase scientific support around sport from different disciplines and perspectives, with the ultimate objective that these studies result in better decision-making in the practical field.

From this editorial, scientists are encouraged to continue focusing their efforts on consolidating the areas of research that have the most outstanding growth, but without forgetting areas of study that do not yet have robust scientific development, and which also need the support provided by empirical data.

## Author Contributions

All authors listed have made a substantial, direct, and intellectual contribution to the work and approved it for publication.

## Conflict of Interest

The authors declare that the research was conducted in the absence of any commercial or financial relationships that could be construed as a potential conflict of interest.

## Publisher's Note

All claims expressed in this article are solely those of the authors and do not necessarily represent those of their affiliated organizations, or those of the publisher, the editors and the reviewers. Any product that may be evaluated in this article, or claim that may be made by its manufacturer, is not guaranteed or endorsed by the publisher.
